# Conformational stability and structural analysis of methanethiol clusters: a revisit†

**DOI:** 10.1039/d1ra04900c

**Published:** 2021-09-01

**Authors:** Manish Kumar Tripathi, Venkatnarayan Ramanathan

**Affiliations:** Department of Chemistry, Indian Institute of Technology (Banaras Hindu University) Varanasi Uttar Pradesh India vraman.chy@iitbhu.ac.in

## Abstract

B3LYP/cc-pV(D/T/Q)Z and CCSD/cc-pVDZ levels of theory predict three minima for both dimers and trimers of methanethiol. Predictions at B3LYP/cc-pVDZ corroborates exceedingly well with the earlier reported experimental value but significantly differ from the previous computational predictions. Interaction energy between the molecules decreases with an increase in the size of the basis set for both the dimer and trimer. The dipole moment of methanethiol dimer gets reduced at the B3LYP/cc-pVDZ level of theory relative to all minima configurations, and the same is seen for trimer also. These new predictions are well supported by atoms in molecules (AIM), frontier molecular orbital (FMO), Mulliken charge (MC), and natural bond orbital (NBO) analysis.

## Abstract 2

B3LYP/cc-pV(D/T/Q)Z and CCSD/cc-pVDZ levels of theory predict three minima for both dimers and trimers of methanethiol.

## Graphical abstract

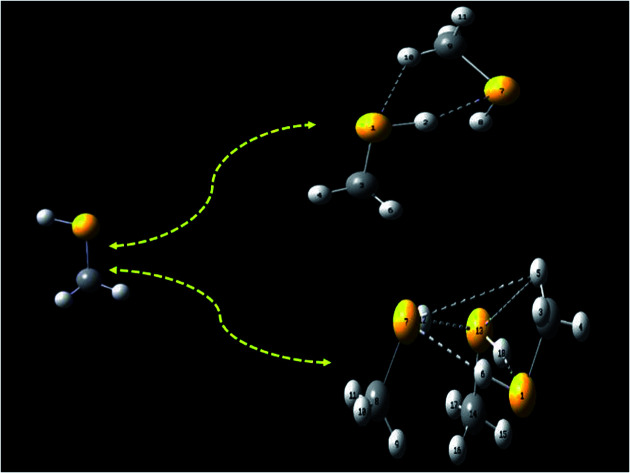

## Introduction

Non-covalent interactions are ubiquitous in several natural processes and play an important role in the cluster formation of molecules.^1^ Generally, interaction energy appears through a non-additive pair-wise donation of electrons. These interactions play a significant role in the strength of the interaction energy, dipole moments, vibrational frequencies, and physical properties of molecular clusters.^2,3^ In order to have a better understanding of the molecular clusters, it is essential to know the interactions in the cluster very accurately. Clusters formed by smaller molecules have been studied extensively in both experiments and computation. Predicting the most stable conformation of a molecular cluster, notwithstanding the size of its constituent, is a non-trivial task. Between the minimum energy conformation and other conformers of the cluster, there exists significant difference in the physical properties of the clusters.^4–6^ Hence the accurate structure of the most stable conformation is of paramount importance as it provides a better understanding towards the conformational analysis.^7^ Knowledge about molecular clusters plays a crucial role in understanding their chemical and biochemical processes resulting in better understanding of non-covalent interactions. There are several reports pertaining to the non-covalent interaction in clusters of water, methanol, ethanol, 1-propanol, methanethiol, ethanethiol, and several other sulfur-containing moieties by *ab initio* methods.^5,8^

The very first study on methanethiol cluster was reported by Sum *et al.*, who reported the conformational structure of methanethiol dimer and trimer.^5^ Without carrying out geometry optimization for methanethiol dimer and trimer, they modeled the structures for these clusters based on their work on the most stable structures of methanol clusters. Subsequently, Cabaleiro-Lago *et al.* carried out an extensive study on methanethiol clusters.^4^ They predicted five minima for both dimer and trimer at the HF, DFT(B3LYP), and MP2 levels of theory using cc-pVDZ and aug-cc-pVDZ basis sets. Amongst the five minima, they identified a global minimum for each dimer and trimer where the methyl hydrogen atom of one molecule interacts with the sulfur atom of the other molecule. In total, there are two such interactions in the dimer. Although Cabaleiro-Lago *et al.* identified a global minimum (structure 2B in ref. 4), among the remaining four minima, there exists another structure (structure 2D in ref. 4) that has similar interaction and energy comparable to that of 2B. Cabaleiro-Lago *et al.* observed this consistently at different levels of theory like HF, MP2, and B3LYP; however, they regarded the predictions at the MP2 level of theory superior to those at HF and B3LYP by stating that these latter two levels of theory underestimate the interaction energy of the dimer. Subsequently, Lung Fu *et al.* carried out time of flight mass spectrometry coupled with IR depletion and vacuum UV ionization studies on methanethiol clusters.^9^ Their experiment revealed only the existence of a single most stable conformation of dimer without giving any information on the structural aspect. In the absence of rotationally resolved studies in their experiment, Lung Fu *et al.* relied completely on Cabaleiro-Lago *et al.*'s prediction at the MP2 level of theory for the conformation of the methanethiol dimer.

It is well documented that the MP2 method neglects electron correlation completely or partially rendering it inappropriate for the study of the non-covalent interactions.^10–14^ Furthermore, as mentioned above regarding the existence of 2D along with 2B, Cabaleiro-Lago *et al.* did not clarify in their work as to why 2D cannot be considered the global minimum along with 2B. Similar kind of problem is also associated with the trimer cluster. Hence there exists a further scope to revisit the predictions of methanethiol clusters, particularly dimer and trimer.

Taking cue of the above-mentioned shortcomings and with an objective of overcoming them, in this work, we revisited the study of non-covalent interactions in methanethiol clusters. We investigate the global minimum conformer of methanethiol dimer and trimer with improved basis sets. With the experimental findings of Lung Fu *et al.* as the benchmark, we also assess the performance of different basis sets and their varying size. Our predictions have better agreement with the experimental results and number of possible minima's of methanethiol cluster.

### Computational detail

Gaussian 16 suite of program was used for all our calculations.^15^ All structures of methanethiol monomer, various conformers of dimers, and trimers were optimized at the B3LYP/cc-pVDZ, cc-pVTZ, &cc-pVQZ CCSD/cc-pVDZ levels of theory. Harmonic vibrational frequency and NBO calculations were also carried out at the CCSD and B3LYP method using the cc-pVDZ basis set for dimer and trimer of methanethiol. Atoms in Molecules (AIM), frontier molecular orbital (FMO) analysis, and Mulliken charge analysis (MCA) were carried out at the B3LYP/cc-pVDZ level of theory. MultiWFN software^16^ was used for AIM calculations. The cluster's optimized energies were corrected for the basis set superposition error (BSSE)^17^ using the Helgonker method,^18^ wherein the correlation energy was extrapolated at the cc-pVNZ (where N = T, Q) for fitting the energies.

1 *E*_corr_ = *a* + *bX*^−3^

where *a* and *b* are the constant parameters to be determined and *X* is a cardinal number, *i.e.*, four for quadruple-zeta sets and five for quintuple-zeta sets.

Methanethiol dimer and trimer were subjected to zero-point energy correction without the use of any scaling factors.

The highest occupied molecular orbital (HOMO) and lowest unoccupied molecular orbital (LUMO) play a crucial role in examining the chemical nature of the molecules. HOMO and LUMO orbitals are responsible for donating electrons and accepting electrons, respectively.^19^ Utilizing results of the FMO analysis for corroboration of the global reactivity descriptors like chemical potential (*μ*), hardness (*η*), electrophilicity index (*ω*), and electronegativity (*χ*) that are obtained by following equations.^20–22^2
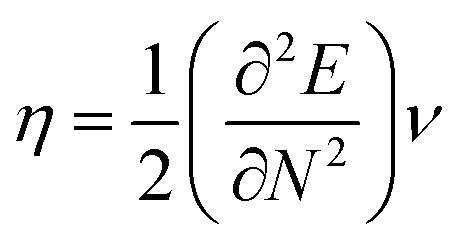
3
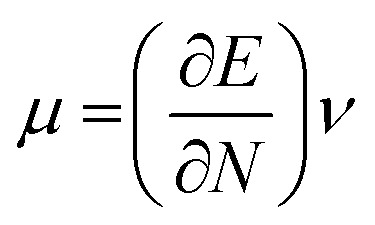
4
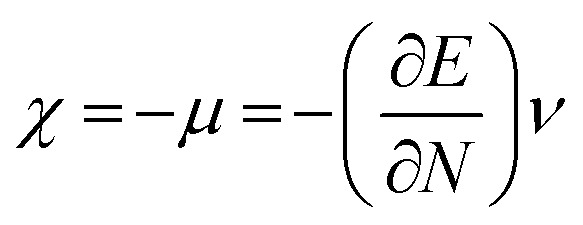
5
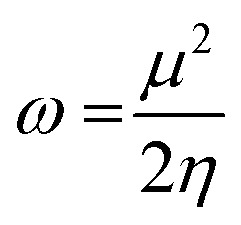
where *E* and *ν* represent the electronic energy and external potential of an *N* electronic system, respectively.

Koopman's theory of closed-shell compound defined *μ*, *η*, and *χ* as follows6
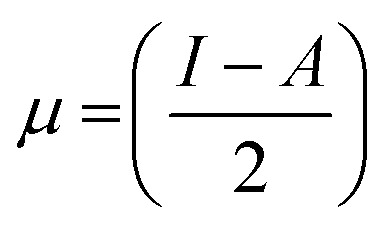
7
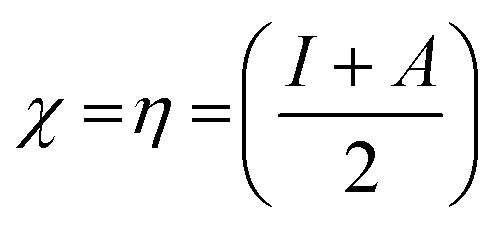
here *A* and *I* represent the ionization potential and electron affinity of the compounds, respectively.

For NBO analysis, the associated stabilization energy *E*^2^ is calculated by using second-order perturbation theory corresponding to each donor NBO_*i*_ and acceptor NBO_*j*_ as.^23^8
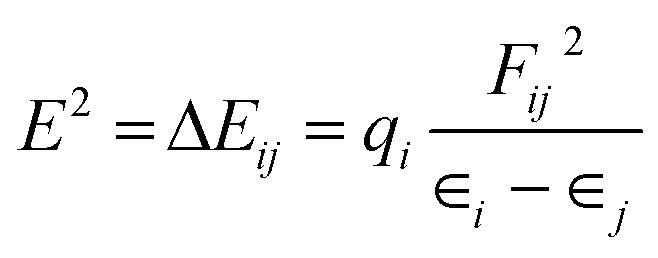
where q_*i*_ is the donor orbital population; *ε*_*i*_, *ε*_*j*_ are orbital energy of the donor and acceptor NBO orbital, respectively; *F*_*ij*_ is the Kohn–Sham matrix element between *i* and *j* NBO orbital's.

## Results and discussion

### Methanethiol dimer

**Fig. 1:**
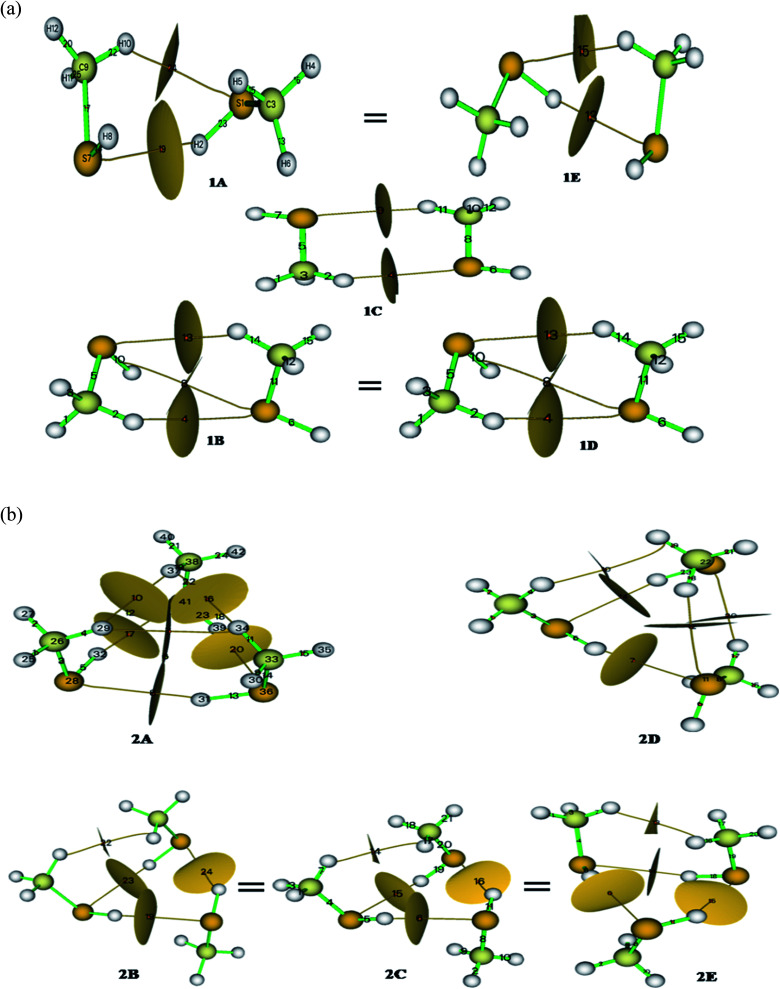
(a) Topological basin surface with bond critical points (3, −1) of methanethiol dimer (B3LYP/cc-pVDZ). (b) Topological basin surface with bond critical points (3, −1) of methanethiol trimer (B3LYP/cc-pVDZ).

Methanethiol dimer and trimer is revisited with contemporarily available basis sets to refine the previously published results and validate experimental findings. The monomer molecule of methanethiol was optimized at different levels of theory. The optimized structure is shown in Fig. SI1,† along with the geometrical parameters predicted from the three basis sets summarized in Table ST1.† In the same table, experimental data is also compared.^24,25^ Table ST1† indicates that there is no significant change in results with higher basis sets. Similar kinds of calculations were carried for methanethiol cluster, which resulted in five conformers (1A, 1B, 1C, 1D, & 1E) having minimum energy and they are shown in (Fig. 1). Conformers 1A & 1E and 1B & 1D have similar types of interaction and have the same energy, and geometrical parameters, which are shown in Tables ST1 and ST2.† Subsequent results from calculations at higher levels of theory were consistent with the DFT method (Tables ST1 and ST2†). In all the five structures, the interaction of the hydrogen atom of a methyl group or sulfur's hydrogen atom with the other molecule of methanethiol was observed.

Cabaleiro-Lago *et al.* identified conformer 1B to be the most stable one. Herein we found that the structures 1A and 1E both conformers have an identical thermodynamic and geometrical parameter with maximum interaction energy and with maximum stability as gleaned from Tables ST2, ST11, and 1.† Conformer 1A or 1E is very similar to the minima of the methanol dimer structure^26^ with the intermolecular distance in methanethiol dimer being greater, *i.e.*, 3.98 Å. This is mainly due to the size of the sulfur atom. The S–H–S–H interaction in conformers 1A, 1B, and 1E, is predominant compared to the interaction C–H–S as shown in Fig. SI2(a)† and 1(a). The most stable conformers 1A or 1E have −4.54 kJ mol^−1^ as interaction energy, whereas the second most stable conformer 1B or 1D have −4.00 kJ mol^−1^ as interaction energy at B3LYP/cc-pVDZ level of theory (refer Tables 1 and ST11†). The interaction energy was seen to decrease with the increasing size of the basis set (−1.875 kJ mol^−1^ at B3LYP/cc-pVQZ) and is shown in Fig. SI3.† These interaction energies are lesser in magnitude when compared to the methanol dimer.^5^

**Table 1:** Relative change in interaction energy of different conformers of dimer and trimer of the methanethiol molecule^a^

	Methanethiol dimer			Methanethiol trimer		
Conformers/methods	1A, 1E	1B, 1D	1C	2B, 2C, 2E	2A	2D
B3LYP/cc-pVDZ	0.00	0.54	1.51	0.00	2.00	2.14
B3LYP/cc-pVTZ	2.67	2.65	3.43	6.22	8.41	7.48
B3LYP/cc-pVQZ	3.24	3.26	3.96	7.38	9.59	8.59
B3LYP/CBS	3.66	3.71	3.96	8.23	10.45	9.40
CCSD/cc-pVDZ	0.78	0.05	0.10	4.80	5.74	3.46

a Note: all values in the above table are referenced with respect to the most stable dimer and trimer at B3LYP/cc-pVDZ as this is the level of theory which matched well with the experiment for normal mode of vibration.

The S–H–S interaction in conformers 1B and 1D is weaker than in conformers 1A, 1C, and 1E; this is due to the methyl group coming closer to the sulfur atom and thereby providing a compact geometry to the dimer of methanethiol. A mentioned before, even though the conformers 1A and 1E have identical energies, their geometrical parameters show slight variation due to electrostatic interactions and inferred by their dipole moments1.8 D & 1.8D (at B3LYP/cc-pVDZ level of theory) for 1A and 1E, respectively. “S–H–S” type interaction is not found in 1B, 1C, and 1D structure of methanethiol dimer, whereas S–H–C type interaction is located at approximately 4.00 Å, and the data is shown in Table ST2.†

Conformer 1C has a C_s_ point group and hence its dipole moment was calculated to be 0.00 D as predicted. Other conformers show deviation from the C_s_ point group attaining C_1_ point group and this happens because of the cooperativity effect of the clustering molecule. Table ST11† shows that the thermodynamic parameter of the dimers at the CCSD/cc-pVDZ level of theory was seen to be slightly higher than the result obtained at the B3LYP/cc-pVDZ level of theory. Contrarily for methanethiol dimer, CCSD method performs poorly compared to the B3LYP level of theory as predictions using the latter method corroborated relatively well with experimental results as shown in Tables 1, ST1, ST9 & ST11.†

### Methanethiol trimer

Out of the five optimized conformers for trimer, three of them namely, 2B, 2C, and 2E attain the global minimum with interaction energies as −15.07 kJ mol^−1^ and −10.27 kJ mol^−1^, respectively at B3LYP/cc-pVDZ and CCSD/cc-pVDZ levels of theory. These three conformers of the trimer have acyclic configuration resulting in an increased number of SH– –S, CH– –S, or CH– –HC interactions as depicted in Fig. SI2(b),†1(b), and Table 1. The difference in the B3LYP and CCSD method is clearly manifested in the calculations pertaining to the trimers, and the same is shown in Fig. SI4.† The value of interaction energy per molecule in the trimer molecule is 4.36 kJ mol^−1^, higher than that of the dimer conformer (2.27 kJ mol^−1^).

Dipole moment of the trimers varies between 1.3 D and 3.8 D (at B3LYP/cc-pVDZ level of theory) with the conformer 2A having the highest value and 2B the lowest. In structure 2A, only S–H–S type interactions take place with an angle 159° at B3LYP and 161° at CCSD levels of theory with cc-pVDZ basis set. In the remaining trimer conformers, other interaction like C–H–S also occur. The numbers of such kinds of interaction in configurations 2B, 2C, 2D, and 2E are more. Conformers 2B, 2C & 2E are much similar with respect to the methyl group's position in cyclic rings of the methanethiol trimer cluster relative to S–H functionality. From Table ST4,† the distance of specific parameters in trimer of methanethiol, it can be seen that the S–H bond is elongated up to 0.006 Å from the monomer and 0.002 Å from the dimer. These variations occurred because of the electronic interactions which promotes the cooperative effect in the hydrogen bonding.

### Normal mode analysis

Generally non-covalent interaction shifts the frequencies of one molecule with the other interacting molecule in the cluster.^27^ Shifting in vibrational frequencies is more prominent because of the hydrogen bonding, whereas the shifting is smaller for vander Waal's interactions. For methanethiol dimer, a shift in the S–H stretching frequency is smaller than the trimer as seen from Tables 2, ST4, ST5 and ST9,† and the largest (red) shift, around 53.8 cm^−1^, occurs in structures 1A or 1E with respect to monomer. Similar variation with different methods, namely B3LYP and CCSD at cc-pVDZ basis set; show blueshifts between 130–78 cm^−1^ (Tables 2 and ST5†). On comparing with experimental vibrational frequency correspond to S–H vibration with the vibrational frequency of the B3LYP/cc-pVDZ functional it was found that one S–H vibrational mode of the dimer is red shifted and other one blue-shifted for most stable conformers 1A or 1E. In contrast, other conformer shows only a blue shift in all S–H vibrational frequencies of the dimer system. Similar calculations were also done for methanethiol trimer, and a significant blue shift (>69.6 but <90 cm^−1^) relative to monomer was observed due to the lengthening in the S–H bond of the methanethiol molecule. Similar variation at other methods was carried out where a blue shift of 90 ± 1 cm^−1^ (refer Table ST5†) was observed. Since other vibrational modes show a minimal frequency shift for dimer and trimer of methanethiol, their impact on the molecule is insignificant, as shown in Table ST6.† The vibrational mode of the hydrogen atom of the methyl group showed almost no significant change. The H_s_SCH_P_ torsion mode was examined following the analysis by Cabaleiro-Lago *et al.*, and observed a red shift of 15 ± 5 cm^−1^ for the dimer and the blue shift of 130 ± 6 cm^−1^ for trimer were observed as depicted in Table ST6.† The calculated vibrational frequencies for monomer, dimer, and trimers of methanethiol molecule corroborates exceedingly well with experimental results^9^ compared to the frequencies computed by Cabaleiro-Lago *et al.* favoring the existence of only three minima for dimer and trimer as against the previously reported five minima.

**Table 2:** Absolute values of the S–H vibrational frequencies (*ν*) at B3LYP/cc-pVDZ level of theory (numbers in bracket represent atom number that are given in Fig. SI2)

	This work	Past work from ref. 9	
S–H stretch	*ν* (cm^−1^)	@MP2/aug-cc-pVDZ (cm^−1^) (Harmonic)^a^	Experiment (cm^−1^)
1A and 1E (7,8)	2600	2749	2601
(1, 2)	2652	2746	
2A (1, 3)	2574	2694	2567
(7, 9)	2584	2674	
(13, 15)	2584	2659	

a Note: the values correspond to the most stable structures (erroneously) assumed by Lung Fu *et al.* in their work.

### Atoms in molecule (AIM) analysis

The distribution of electron densities into molecules is the source of the various properties and forces experienced by the molecule or molecular systems. Electron density and Laplacian electron density helps in the fair assessment of the molecular interaction (ether interaction is a covalent or non-covalent type). The plethora of studies validate the characterization of the non-covalent interaction by the electron density *ρ*(*r*) and Laplacian electron density ▽^2^*ρ*(*r*); hence we carried out AIM calculations^28^ for our system to elucidate the actual nature of the interactions. According to Koch and Popelier, bond critical points (BCP) decides the path of the hydrogen bond (H– acceptor)^29^ that has electron density in the range of 0.002–0.040 a.u. and Laplacian electron density in the range of the 0.024–0.139 a.u. Calculated parameters of the AIM analysis at BCP are summarized in Table ST10,† and the corresponding BCP with basin surfaces are given in Fig. 1.

No of (3, −1) BCPs are same for iso-energy conformers (for dimer 1A = 1E & 1B = 1D; for trimer 2B = 2C = 2E) in both kind of clusters. Topological parameters of the BCPs validate the existence of the three different kinds of hydrogen bonds. For this, Rozas *et al.* set three conditions based on the Laplacian electron density (∇^2^*ρ*); for strong and covalent nature of the hydrogen bonds ∇^2^*ρ* <0 and H <0, for medium and partially covalent nature of the hydrogen bond ∇^2^*ρ* >0 and H <0, and for weak hydrogen bond ∇^2^*ρ* >0 and H >0. From Table ST10,† it can be seen that the values of ∇^2^*ρ* and H are positive for methanethiol dimer, implying a weak interaction. In contrast, methanethiol trimer has positive values of ∇^2^*ρ* and H corresponding to conformer 2A, 2B, 2C, 2E and reversal in the sign is observed for conformer 2D, which signifies that the conformers 2A, 2B, 2C, & 2E have a weak interaction and conformer 2D has a strong interaction. For these, interactions we calculated interaction energies by adopting Espinosa *et al.* predictions;^30^ by solving the equation (*E*_int_ = ½*V*) at BCP. Higher values of interaction energy were obtained for dimer 1A and 1E and trimer 2D conformers. Thus based on AIM calculation, it can be concluded that conformers 1A and 1E are the stable configurations for methanethiol dimer and these are thermodynamic and kinetically favorable conformers, which is in contrast to the predictions by Cabaleiro-Lago *et al.* who identified 1B as the stable one. In the case of the trimer too, it is found that 2E, 2C, and 2B are the most stable as against the earlier report by Cabaleiro-Lago *et al.* who identified 2C to be the most stable one. These predictions have good agreement with the thermodynamic parameter analysis, FMO, MCA, and NBO analysis.

Summary of the computed energy parameter of methanethiol dimer and trimer at CCSD/cc-pVDZ level of theory

**Table:** 

Dimer			
Energy parameter	1A & 1E	1B & 1D	1C
*E*_HOMO_ (IP) (kcal mol^−1^)	−220.7	−221.0	−221.8
*E*_LUMO_ (EA) (kcal mol^−1^)	86.6	90.9	91.0
HOMO–LUMO gap (*E*_g_)	307.3	311.9	312.8
Dipole moment (D)	1.82	1.47	0.00
Hardness (*η*)	−67.1	−65.1	−65.4
Chemical potential (*μ*)	67.1	65.1	65.4
Electronegativity (*χ*)	−67.1	−65.1	−65.4
Electrophilicity index (*ω*)	−33.6	−32.6	−32.7

**Table:** 

Trimer			
	2A	2B, 2C, & 2E	2D
*E*_HOMO_ (IP) (kcal mol^−1^)	−222.7	−222.6	−222.5
*E*_LUMO_ (EA) (kcal mol^−1^)	78.9	89.5	89.5
HOMO–LUMO gap (*E*_g_)	301.6	312.1	312.0
Dipole moment (*D*)	3.43	1.40	1.80
Hardness (*η*)	−71.9	−66.6	−66.5
Chemical potential (*μ*)	71.9	66.6	66.5
Electronegativity (*χ*)	−71.9	−66.6	−66.5
Electrophilicity index (*ω*)	−36.0	−33.3	−33.3

### Frontier molecular orbital (FMO) analysis

Energy profile diagrams of the HOMO and LUMO orbitals are given in Fig. SI5 and SI6.† All calculated energy parameters of FMOs are shown in Table 3.

HOMO–LUMO gap (H–L gap) defines the molecule's chemical hardness which in turn indicates the thermodynamic stability of the system. Larger the H–L gap, the harder is the system or in other words, more stable and *vice versa*. The H–L gap (*E*_g_) for the most stable conformer of the dimer (1A or 1E) is 307.3 kcal mol^−1^, and for the trimer, 2B or 2C or 2E is 312.1 kcal mol^−1^. The H–L gap is more for the trimer, implying that the trimer is thermodynamically more stable compared to the dimer.

### Mulliken charge analysis (MCA)

Mulliken charges (MC) are the essential fundamental tool for describing the process of electronegativity and for the transfer of charges in the molecules. It depends on the vibrational modes of the molecule which is responsible for the changes in the dipole moment, electronic structure, polarizability, and strength of the hydrogen bond.^31^ MCA calculation was done for all minima of dimer and trimer of methanethiol, and the corresponding results are given in Table 4.

Mulliken charge on the atoms into the methanethiol dimer and trimer [CCSD/cc-pVDZ] (values are expressed in atomic units)

**Table:** 

Dimer			
Atom label	1A and 1E	1B and 1D	1C
1(S)	−0.112	−0.106	−0.105
2(H of S)	0.083	0.075	0.065
3(C)	−0.175	−0.180	−0.180
4(H of C)	0.072	0.078	0.067
5(H of C)	0.058	0.070	0.086
6(H of C)	0.070	0.069	0.068
7(S)	−0.099	−0.110	−0.105
8(H of S)	0.066	0.066	0.065
9(C)	−0.183	−0.180	−0.181
10(H of C)	0.076	0.068	0.086
11(H of C)	0.075	0.085	0.067
12(H of C)	0.070	0.065	0.068

**Table:** 

Trimer			
Atom label	2A	2B, 2C, and 2E	2D
1(S)	−0.114	−0.122	−0.122
2(C)	−0.181	−0.176	−0.176
3(H of S)	0.089	0.074	0.089
4(H of C)	0.075	0.074	0.074
5(H of C)	0.072	0.067	0.067
6(H of C)	0.061	0.089	0.074
7(S)	−0.114	−0.115	−0.127
8(C)	−0.181	−0.182	−0.173
9(H of S)	0.089	0.063	0.089
10(H of C)	0.075	0.074	0.073
11(H of C)	0.072	0.071	0.073
12(H of C)	0.061	0.085	0.065
13(S)	−0.114	−0.127	−0.116
14(C)	−0.181	−0.173	−0.182
15(H of S)	0.089	0.073	0.085
16(H of C)	0.075	0.065	0.074
17(H of C)	0.072	0.073	0.071
18(H of C)	0.061	0.089	0.063

Table 4 shows that the MC on sulfur and carbon atom contains negative charges, and the hydrogen atom bears a positive charge. In both cases, sulfur contains a more negative charge relative to the carbon atom, and the hydrogen atom that is connected to sulfur has a higher positive charge. The hydrogen atom of the carbon atom has a slightly smaller positive charge. This is because of the delocalization of the charge intra-molecularly. MC of sulfur and hydrogen in trimers are slightly decreased because of the increased interaction in trimer. These results strongly reinforce the predictions of the FMO and AIM calculations described above and NBO analysis shown below.

### Natural bonds orbital (NBO) analysis

NBO analysis is an accurate method for understanding inter and intra molecular interactions and the extent of charge transfer in a molecular system.^31^ NBO analysis gives second order perturbation energy [Interaction energy *E*^2^] which is responsible for the strength of the respective interactions. As *E*^2^ increases, the interaction between donor and acceptor orbital increases, and consequently strength of the interaction also increases. Results of the NBO analysis are summarized in Table ST8.† From Table ST8,† it is inferred that the trimer has more interactions compared to the dimer. This once again indicates that the trimer is more stable compared to the dimer. Overlapping of orbital *n*(S) lone pair of one molecule with σ*_S–H_ molecular orbital (MO) of the other molecule helps in the generation of intermolecular hydrogen bonding (IHB), which was observed in dimer and trimer as depicted in Table ST8.† Due to these interactions, some electrons get transferred, and these are responsible for the increment of electron density in orbital σ*_S–H_ (ABMO), and the strength of the σ_S–H_ [bonding molecular orbital (BMO)] bond is slightly reduced. In the case of intra-molecular interaction, the only filled orbitals σ_S–H_ and lone pair LP(2) can overlap with σ*_C–H_ bond, which also weakens the strength of the σ_C–H_ bond. They have a significant role in the stabilization of the cluster. The changes in the energies corresponding to the Lewis base and Lewis acid sites, which strongly favors the existence of inter and intramolecular interactions in methanethiol cluster and clustering, is possible only on the expenses of these kinds of interactions.

The second-order perturbation energy (stabilization energy) *E*^2^ associated with hyperconjugation correspond to Lewis base-Lewis acid sites into the same molecule or with other molecules are summarized in Table ST8.† The variations into *E*^2^ energies are mainly because of the accumulation of electron density into σ*_C–H_ bond, and σ*_S–H_ molecular orbitals is not only drawn from *n*(*S*) of hydrogen acceptor but also from σ_S–H_ molecular orbital.

So from the above discussions, we can say that the geometrical parameter of dimer and trimer is decreased corresponding to the S–C bond, and the bond length of the S–H bond is slightly increased in the dimer, but in the case of the trimer, it is decreased. Variation in H–C–H angle is because of the crowding and for attaining a stable conformation. Thus, the similarity of the conformer 1A with 1E, 1B with 1D, and 2B, with 2C & 2E, are well verified by NBO results very well. These conformers have similar interactions and the same number of interaction ref Fig. 1 and SI2.† These predictions are well supported by AIM, FMO, MCA, and vibrational analysis.

## Conclusion

In this work, we performed a computational study to investigate structures with minimum energy for the methanethiol molecular system by using B3LYP with cc-pVDZ, cc-pVTZ, and cc-pVQZ basis sets. Further, we extended our calculations at a higher level of the method, *i.e.*, the CCSD method with a cc-pVDZ basis set. We found that the interaction energy, dipole moment, and geometrical parameter have the same values for the CCSD and B3LYP methods with the cc-pVDZ basis set. Thus it is concluded that there are three minima in place of five for both dimer and trimer of methanethiol. Among the three minima of the dimer, 1A and 1E are identified as the most stable ones as against the earlier prediction by Cabaleiro-Lago *et al.*, who identified 1B to be the most stable one. In the case of the trimer, it is concluded that there are three minima where 2E, 2C, and 2B are the most stable ones, contrary to Cabaleiro-Lago *et al.*’s report where they identified only 2C to be the most stable one. The mode of interactions between the trimer's minima is slightly different relative to the dimer conformers. Configurations of trimers that exhibit S–H–S type interaction along with the interaction between sulfur and methyl group is responsible for higher stabilization because of the lesser electronic repulsion (between the non-bonded e^−^ of the sulfur atom). These findings are well supported by the vibrational, AIM, FMO, MC, and NBO results that have better agreement with experimental results.

## Conflicts of interest

There are no conflicts to declare.

## Supplementary Material

RA-011-D1RA04900C-s001
