# Supplementary material for: Conformational stability and structural analysis of methanethiol clusters: a revisit
Source: RSC Adv. 2021 Sep 1;11(47):29207–14. doi: 10.1039/d1ra04900c (PMC9040644; doi:10.1039/d1ra04900c)
Supplement: RA-011-D1RA04900C-s001 [file RA-011-D1RA04900C-s001.pdf]

Electronic Supplementary Material (ESI) for RSC Advances.
This journal is © The Royal Society of Chemistry 2021

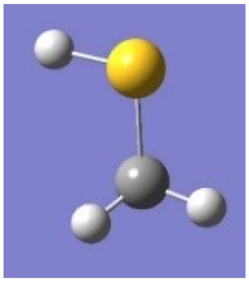

Fig.SI1 Optimized geometry of methanethiol monomer at B3LYP/cc-pVDZ level of theory

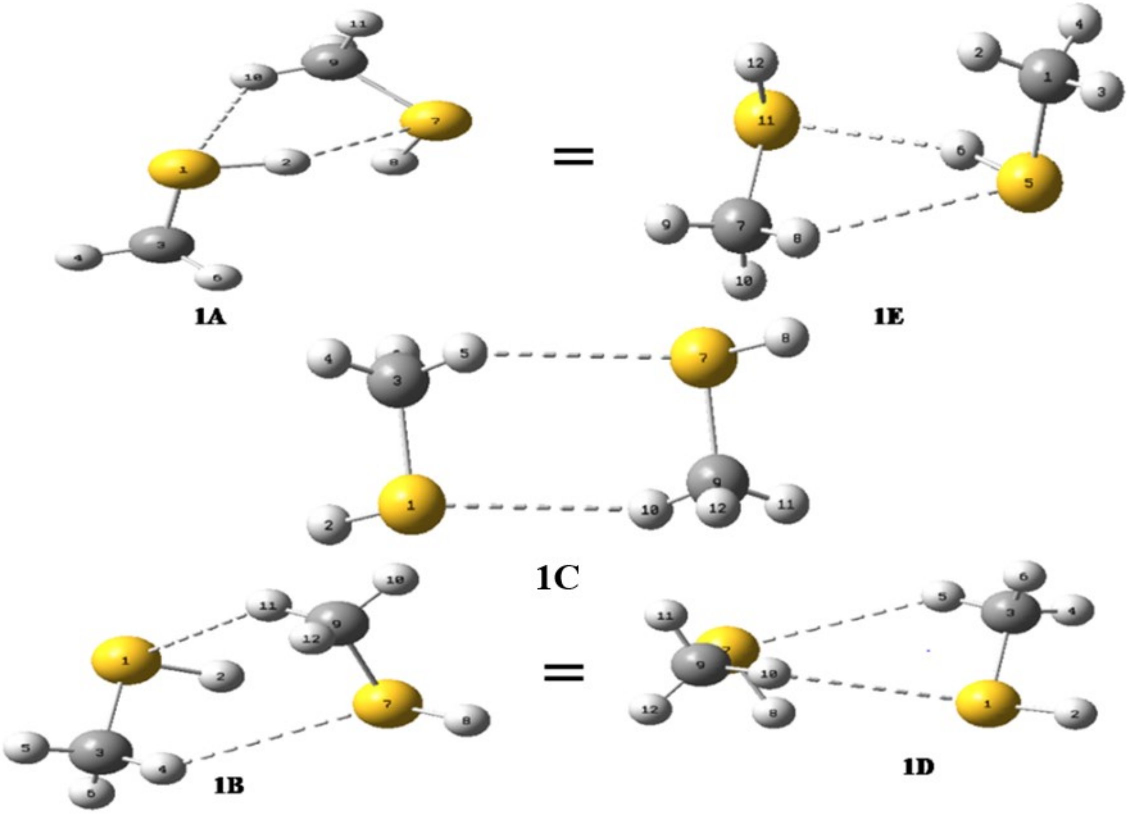

Fig. SI 2(a). Optimized geometry of the conformers of methanethiol dimer at B3LYP/cc-pVDZ

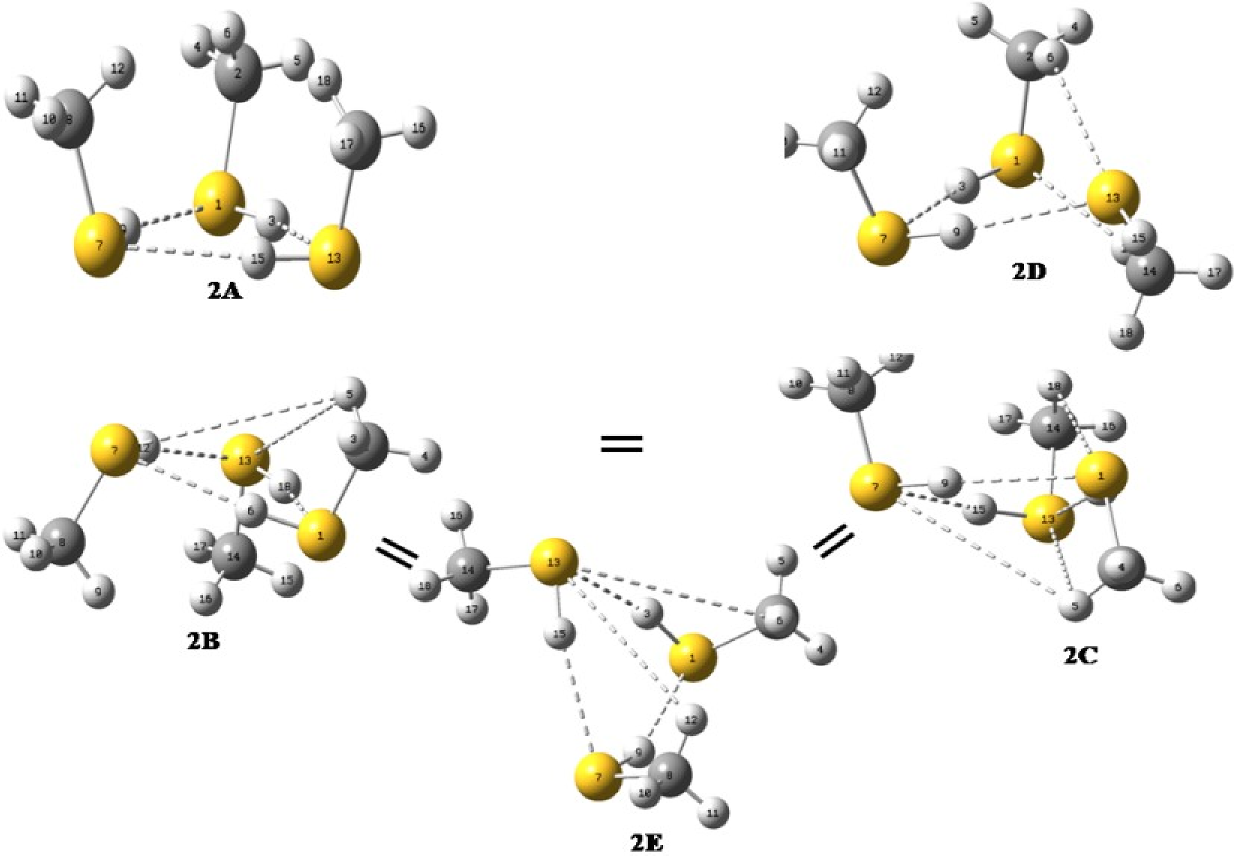

Fig. SI 2(b). Optimized geometry of the conformers of methanethiol trimer at B3LYP/cc-pVDZ

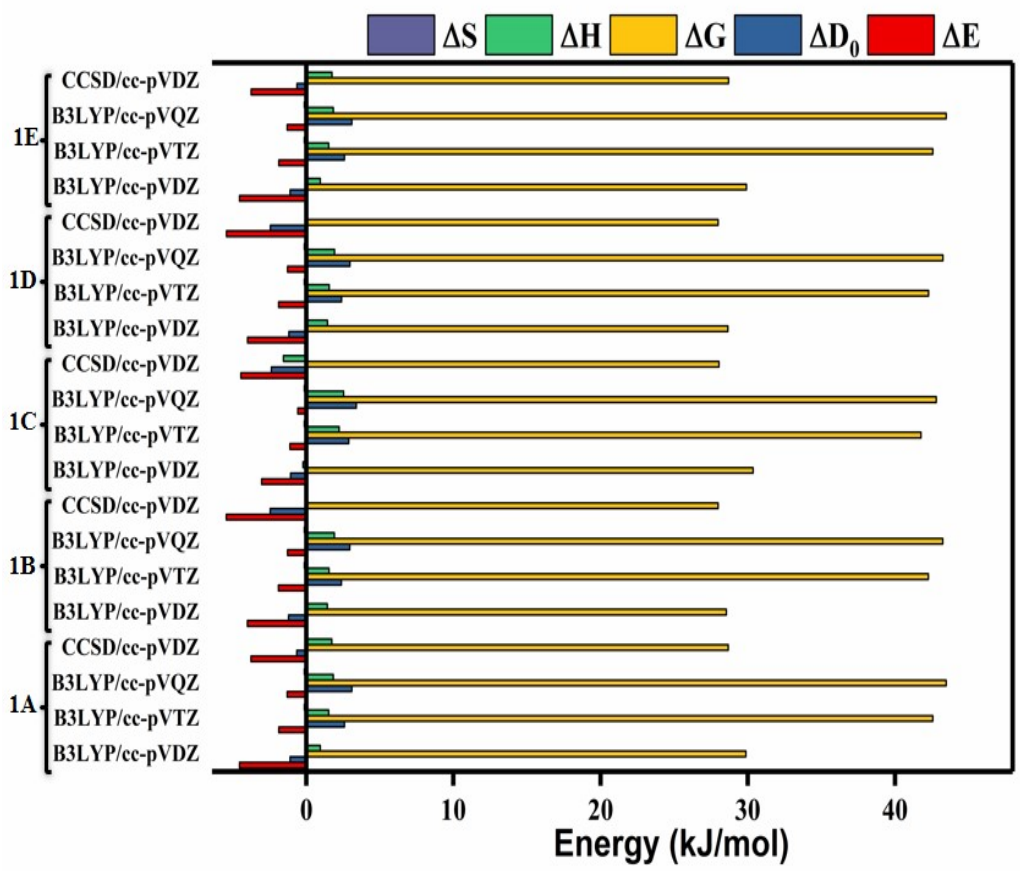

Fig. SI 3. Counterpoise corrected thermodynamic parameter of methanethiol dimer at various levels of theory and different basis sets.

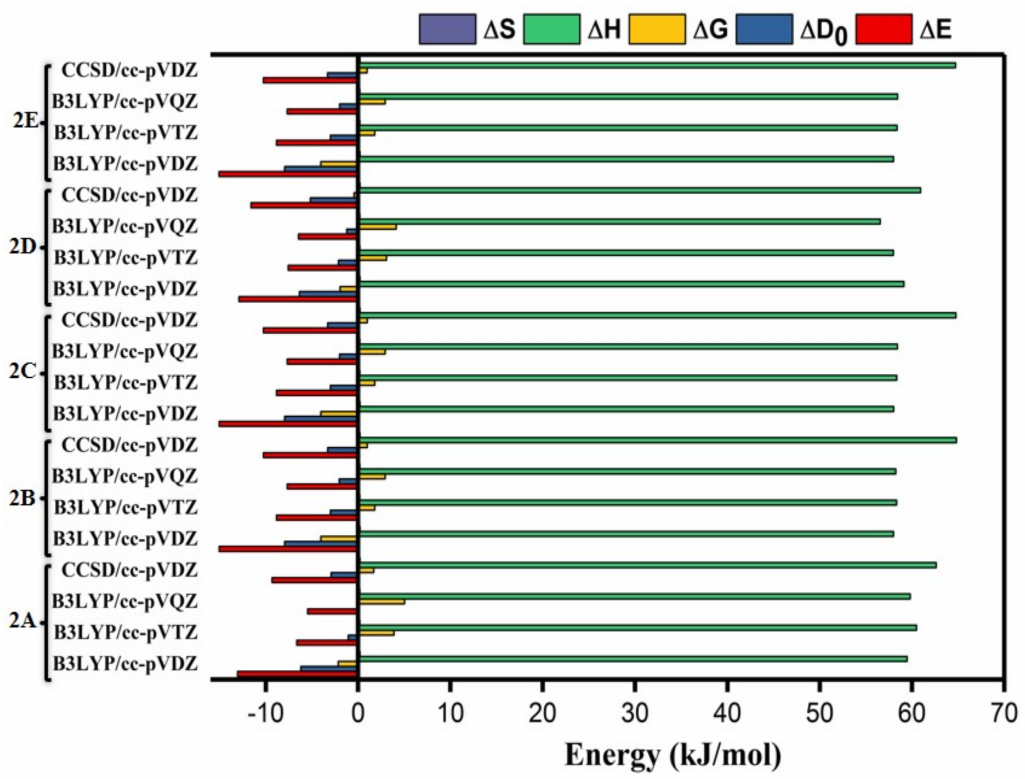

Fig. SI 4. Counterpoise corrected thermodynamic parameter of methanethiol trimer at various levels of theory and different basis sets.

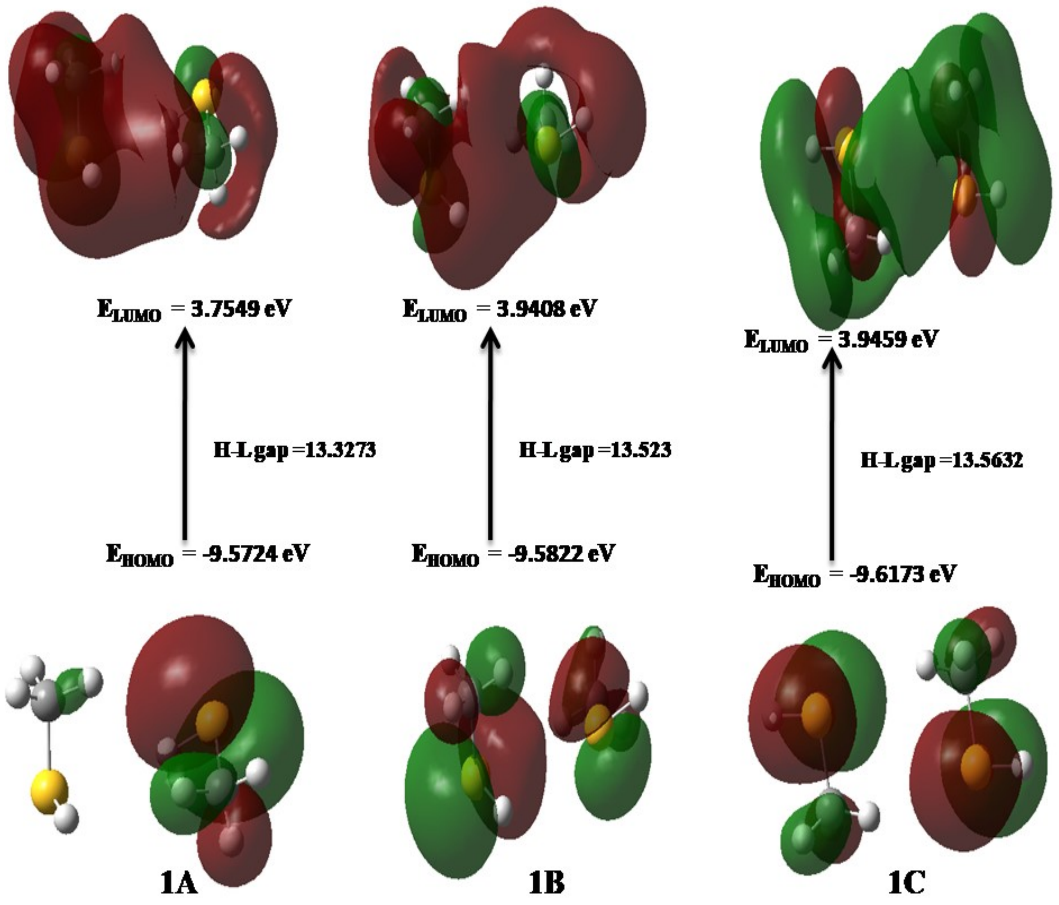
E$_{LUMO}$ = 3.7549 eV
E$_{LUMO}$ = 3.9408 eV
E$_{LUMO}$ = 3.9459 eV
H-L gap =13.3273
H-L gap =13.523
H-L gap =13.5632
E$_{HOMO}$ = -9.5724 eV
E$_{HOMO}$ = -9.5822 eV
E$_{HOMO}$ = -9.6173 eV
1A
1B
1C

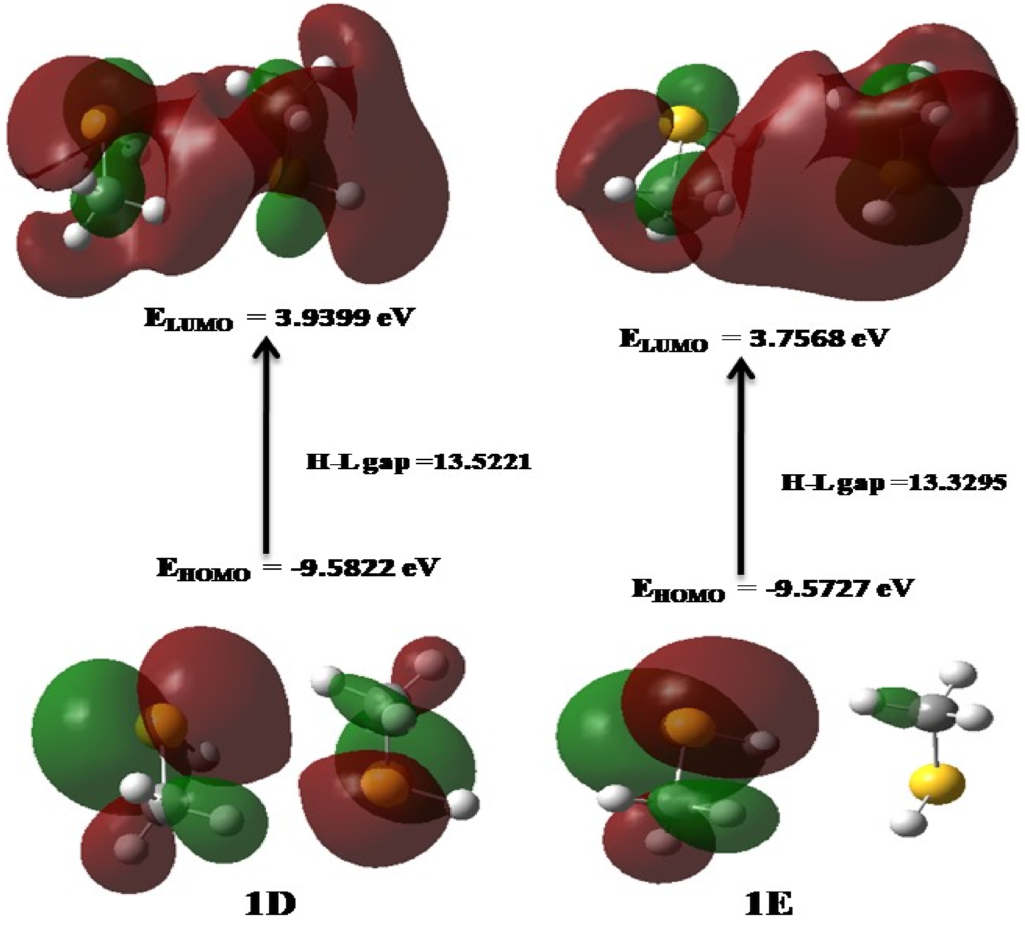

Fig. SI 5. FMO isosurface of methanethiol's dimer.

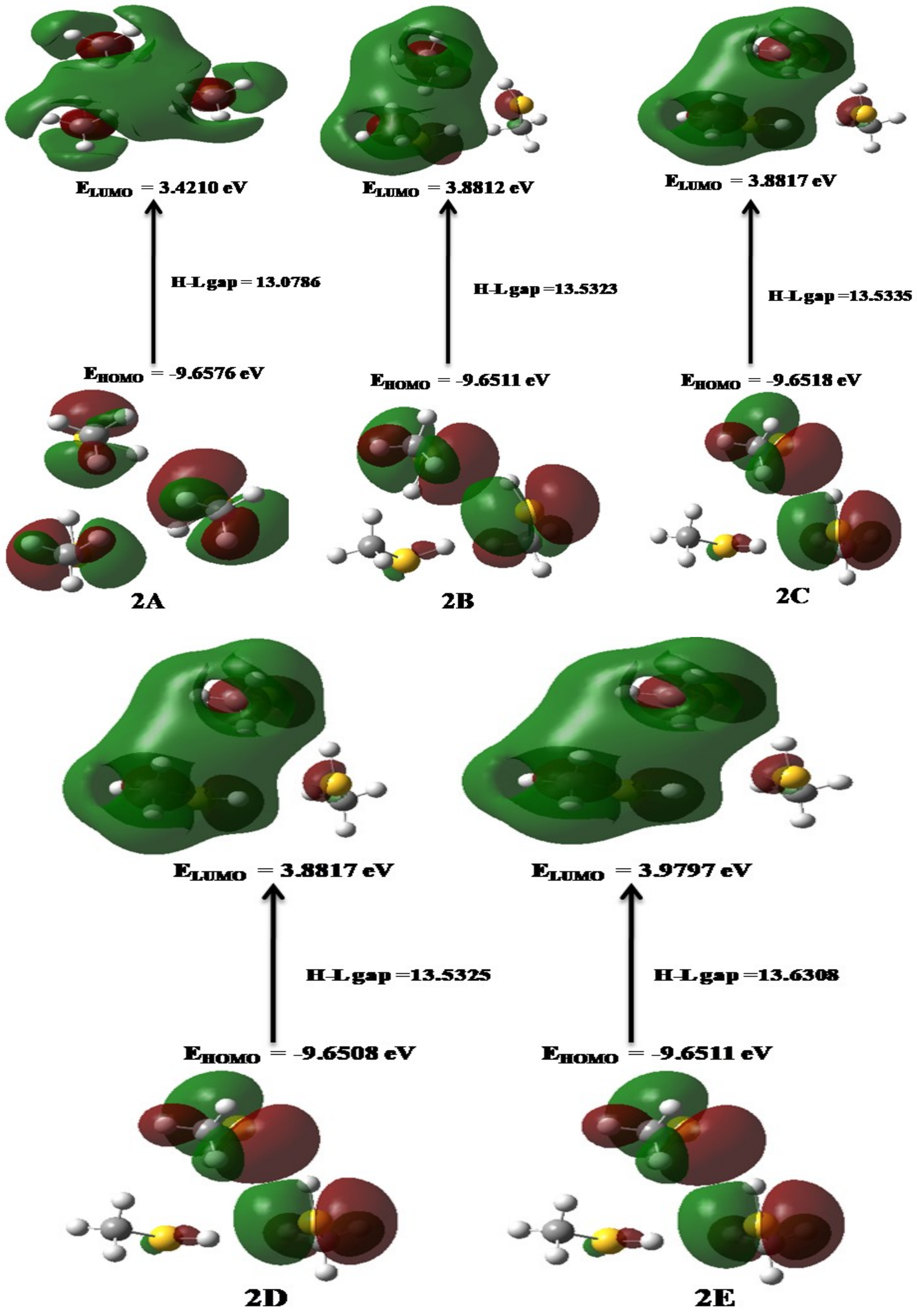

Fig. SI 6. FMO iso-surface of methanethiol trimer

Table: ST1. Optimized geometrical parameters of the methanethiol monomer (R is distance in Å, Θ is angle in degree (°) and μ is dipole moment in D)

	Our calculation at B3LYP			Lago et al's calculation	Experimental Values
	cc-pVDZ	cc-pVTZ	cc-pVQZ	B3LYP/cc-pVDZ	[From Ref. 24, 25.]
$R_{s\text{-}c}$	1.84	1.83	1.83	1.84	1.82
$R_{S\text{-}H}$	1.36	1.34	1.34	1.36	1.33
$R_{C\text{-}H}$	1.10	1.09	1.09	1.10	1.10
$R_{C\text{-}H}$	1.10	1.09	1.09	1.10	1.10
$\Theta_{H\ s\text{-}S\text{-}C}$	96.9	97.1	97.3	97.0	100.3
$\Theta_{H\text{-}C\text{-}H}$	108.8	108.9	108.8	109.0	110.3
$\Theta_{H\text{-}C\text{-}H}$	110.3	110.4	110.4	110.5	110.3
μ	1.50	1.55	1.55	1.61	1.52

Table: ST 2. Optimized geometrical parameters of dimers of methanethiol at B3LYP (R and r* are distances in Å, Θ is angle in degree (°) and μ is dipole moments in D))

	1A			1B			1C			1D			1E		
	cc-pVDZ	cc-pVTZ	cc-pVQZ	cc-pVDZ	cc-pVTZ	cc-pVQZ	cc-pVDZ	cc-pVTZ	cc-pVQZ	cc-pVDZ	cc-pVTZ	cc-pVQZ	cc-pVDZ	cc-pVTZ	cc-pVQZ
$R_{C\text{-}S1}$	1.84	1.84	1.84	1.84	1.84	1.84	1.84	1.84	1.84	1.84	1.84	1.84	1.84	1.84	1.84
$R_{H\text{-}S1}$	1.36	1.36	1.36	1.36	1.36	1.36	1.36	1.36	1.36	1.36	1.36	1.36	1.36	1.36	1.36
$R_{C\text{-}S7}$	1.84	1.84	1.84	1.84	1.84	1.84	1.84	1.84	1.84	1.84	1.84	1.84	1.84	1.84	1.84
$R_{H\text{-}S7}$	1.36	1.36	1.36	1.36	1.36	1.36	1.36	1.36	1.36	1.36	1.36	1.36	1.36	1.36	1.36
$\Theta_{C\text{-}S1...S}$	98.1	98.1	98.1	77.2	77.2	77.2	66.4	66.4	66.4	75.1	75.1	75.1	98.3	98.3	98.3
$\Theta_{C\text{-}S7...S}$	75.8	75.8	75.8	78.3	78.3	78.3	66.3	66.3	66.4	77.3	77.3	77.3	75.9	75.9	75.9
$\Phi_{C\text{-}S...S\text{-}C}$	123.0	123.0	123.0	-116.9	-116.9	-116.9	180.0	180.0	180.0	-116.8	-116.8	-116.8	-123.7	-123.7	-123.7
$r^*_{S...S}$	4.06	4.06	4.06	3.98	3.98	3.98	4.41	4.41	4.41	3.98	3.98	3.98	4.06	4.06	4.06
$r^*_{H...S1}$	3.22	3.22	3.22	3.14	3.14	3.14	4.13	4.13	4.13	3.17	3.17	3.17	3.23	3.23	3.23
$r^*_{H...S7}$	2.80	2.80	2.80	3.17	3.17	3.17	4.13	4.13	4.13	3.14	3.14	3.14	4.36	4.36	4.36
$\Theta_{X\text{-}H...S1}$	130.4	130.4	130.4	139.1	139.1	139.1	77.8	77.8	77.8	132.9	132.9	132.9	130.3	130.3	130.3
$\Theta_{X\text{-}H...S7}$	152.5	152.5	152.5	133.1	133.1	133.1	77.8	77.8	77.8	139.0	139.0	139.0	100.7	100.7	100.7
μ	1.82	1.79	1.77	1.47	1.48	1.48	0.00	0.00	0.00	1.48	1.49	1.48	1.81	1.78	1.76

Table: ST 3. Optimized geometrical parameters of the methanethiol trimer (R and r are distances in Å, Θ is angle in degree (°) and μ is dipole moments in D))

Geometrical Parameters	2A		2B		2C		2D		2E	
	CCSD	B3LYP	CCSD	B3LYP	CCSD	B3LYP	CCSD	B3LYP	CCSD	B3LYP
R_{S-H}	1.35	1.36	1.35	1.36	1.35	1.36	1.35	1.36	1.35	1.36
	1.35	1.36	1.35	1.36	1.35	1.36	1.35	1.36	1.35	1.36
	1.35	1.36	1.35	1.36	1.35	1.36	1.35	1.36	1.35	1.36
$r_{H...S1}$	2.89	2.77	2.85	2.73	2.85	2.73	3.03	3.01	3.00	2.77
	-	-	-	-	3.35	4.25	-	-	-	-
$r_{H...S7}$	2.89	2.77	2.89	4.42	2.99	2.80	2.94	2.77	2.90	2.73
	-	-	3.82	2.73	3.24	4.30	-	-	-	-
$r_{H...S13}$	2.89	2.77	2.99	2.77	-	2.73	2.82	2.74	2.85	4.43
	-	-	3.24	4.30	3.83	4.42	3.11	3.46	4.94	4.26
	-	-	-	-	2.90	-	-	-	3.35	2.73
$\Theta_{X-H...S1}$	158.6	160.8	153.2	161.7	129.2	161.7	154.3	156.1	145.3	159.7
	-	-	-	-	153.3	111.1	-	-	-	-
$\Theta_{X-H...S7}$	158.6	160.7	112.7	97.3	126.5	159.6	149.2	165.6	144.9	161.6
	-	-	144.9	161.5	145.5	110.2	-	-	-	-
$\Theta_{X-H...S13}$	158.6	160.8	126.4	159.7	144.9	161.6	149.2	163.5	90.2	97.2
	-	-	145.4	110.2	112.6	97.2	149.2	139.5	153.2	161.7
	-	-	-	-	-	-	-	-	129.2	111.0
μ	3.43	3.75	1.40	1.25	1.40	1.25	1.80	2.20	1.40	1.25

Table: ST 4 Selected optimized geometrical parameters of methanethiol monomer, its most stable dimer (1A&1E) and its trimer (2B, 2C and 2E) (R is distance in Å, Θ is angle in degree (°) and μ is dipole moments in D))

Geometrical Parameters	B3LYP/cc-pVDZ		
	Monomer	Dimer	Trimer
R_{S-C}	1.837	R_{S1-C} = 1.83	R_{S1-C2} = 1.83
		R_{S7-C} = 1.84	R_{S7-C8} = 1.83
			$R_{S13-C14}$ = 1.83
R_{S-H}	1.358	R_{S1-H} = 1.36	R_{S1-H3} = 1.35
		R_{S7-H} = 1.36	R_{S7-H9} = 1.35
			$R_{S13-H15}$ = 1.35
R_{C-H}	1.099	R_{C3-H4} = 1.10<br>R_{C3-H5} = 1.10<br>R_{C3-H6} = 1.10	R_{C2-H4} = 1.10<br>R_{C2-H5} = 1.10<br>R_{C2-H6} = 1.10
		R_{C9-H10} =1.10<br>R_{C9-H11} =1.10<br>R_{C9-H12} =1.10	R_{C8-H10} = 1.10<br>R_{C8-H11} = 1.10<br>R_{C8-H12} = 1.10
			$R_{C14-H16}$ = 1.10<br>$R_{C14-H17}$ = 1.10<br>$R_{C14-H18}$ = 1.10
Θ_{H-C-H}	108.77	$\Theta_{4, 3, 5}$ =35.6<br>$\Theta_{4, 3, 6}$ =108.8<br>$\Theta_{6, 3, 5}$ =109.8	$\Theta_{2, 4, 5}$ = 35.6<br>$\Theta_{4, 2, 6}$ = 108.8<br>$\Theta_{2, 6, 5}$ =110.0
		$\Theta_{12, 9, 11}$ =108.7<br>$\Theta_{10, 9, 11}$ =108.8<br>$\Theta_{12, 9, 10}$ =110.6	$\Theta_{10, 8, 11}$ =108.7<br>$\Theta_{10, 8, 12}$ =108.9<br>$\Theta_{12, 8, 11}$ =110.0
			$\Theta_{17, 14, 18}$ = 109.8<br>$\Theta_{17, 14, 16}$ = 108.9<br>$\Theta_{16, 14, 18}$ = 108.9
$\Theta_{H-S-C-H}$	180	$\Theta_{H2-S1-C3-H4}$ = -177.6	$\Theta_{H3-S1-C2-H4}$ = 179.2
		$\Theta_{H8-S7-C9-H11}$ =-177.6	$\Theta_{H9-S7-C8-H10}$ = 177.6
			$\Theta_{H15-S13-C14-H16}$ = -175.1
μ	1.50 D	1.71 D	1.40 D

Table: ST 5. Relative change in vibrational frequency (Δυ) and intensity (**I**) of S-H bonds in dimer and trimer of methanethiol with respect to monomer ($\mathbf{I_o}$) (Δυ in cm^{-1})

	B3LYP/cc-pVDZ		CCSD/ cc-pVDZ	
	Δυ	I/I_o	Δυ	I/I_o
**1A (7,8)**	-53.92	4.66	-3.81	0.63
**(1, 2)**	-1.10	0.82	-3.23	0.57
**1B (7,8)**	-2.83	1.47	-1.55	1.38
**(1,2)**	2.02	0.43	1.12	0.40
**1C (7,8)**	-2.12	2.20	-2.03	2.19
**(1, 2)**	-2.06	0.00	-2.02	0.00
**1D (7,8)**	-3.05	1.47	-1.63	1.38
**(1, 2)**	2.08	0.43	1.00	0.40
**1E (12, 11)**	-53.81	4.66	-3.76	0.64
**(6, 5)**	-1.12	0.82	-3.23	0.56
**2A (1, 3)**	-79.07	0.05	-14.87	0.05
**(7, 9)**	-69.60	12.73	-10.54	1.34
**(13, 15)**	-68.96	12.71	-10.51	1.33
**2B (1, 3)**	-90.88	1.80	-13.88	0.55
**(13, 15)**	-80.98	15.59	-6.46	0.44
**(7, 9)**	-90.92	12.00	-4.89	0.35
**2C (7, 9)**	-90.92	1.82	-14.03	0.56
**(1, 3)**	-81.01	15.57	-6.54	0.45
**(13, 15)**	-72.65	11.99	-4.91	0.35
**2D (7, 9)**	-72.73	4.95	-12.79	1.00
**(1, 3)**	-66.08	11.86	-5.59	0.51
**(13, 15)**	-0.73	0.91	-2.36	0.96
**2E (1,3)**	-90.79	1.80	-14.01	0.56
**(13, 15)**	-80.88	15.55	-6.46	0.45
**(7, 9)**	-72.64	12.01	-4.81	0.34

1. All frequencies are calculated by subtracting the frequency of dimer/trimer from the frequency of the monomer for the corresponding vibrational mode.
2. I is the computed IR intensity of the dimer/trimer and I_o is the computed IR intensity of the monomer.

Table: ST 6. Relative change in torsion frequency HSCH in dimer and trimer of the methanethiol with respect to monomer

	B3LYP		CCSD	
	**Δυ**	**I/I_o**	**Δυ**	**I/I_o**
**1A (8, 7, 9, 11)**	26.13	1.75	17.23	1.42
**(2, 1, 3, 4)**	121.71	1.90	79.36	1.92
**1B (8, 7, 9, 11)**	15.38	1.73	14.13	1.56
**(2, 1, 3, 4)**	19.79	1.06	17.43	1.21
**1C (8, 7, 9, 11)**	4.77	0.00	6.90	0.00
**(2, 1, 3, 4)**	13.87	1.70	13.10	1.80
**1D (8, 7, 9, 11)**	15.63	1.78	14.43	1.58
**(2, 1, 3, 4)**	20.21	1.02	17.48	1.19
**1E (12, 11, 7, 10)**	26.1	1.75	18.23	1.41
**(6, 5, 1, 4)**	121.59	1.90	79.25	1.93
**2A (9, 7, 8, 12)**	-135.99	0.10	-142.95	0.26
**(3, 1, 2, 5)**	-135.62	0.10	-142.83	0.26
**(15, 13, 14, 17)**	-121.87	0.00	-126.64	0.01
**2B (3, 1, 2, 5)**	-135.97	0.09	-148.65	0.19
**(9, 7, 8, 12)**	-134.83	0.10	-132.64	0.19
**(15, 13, 14, 17)**	-122.49	0.00	-108.78	0.21
**2C (9, 7, 8, 11)**	-136.01	0.09	-148.55	0.19
**(3, 1, 2, 5)**	-134.78	0.09	-132.86	0.19
**(15, 13, 14, 17)**	-122.48	0.00	-109.01	0.21
**2D (9, 7, 8, 12)**	-148.02	0.04	-161.61	0.13
**(3, 1, 2, 5)**	-124.65	0.11	-137.11	0.29
**(15, 13, 14, 17)**	-115.89	0.06	-116.9	0.12
**2E (9, 7, 8, 12)**	-136.02	0.10	-148.54	0.19
**(3, 1, 2, 5)**	-134.79	0.10	-132.89	0.19
**(15, 13, 14, 17)**	-122.48	0.00	-109.51	0.20

3. All frequencies are calculated by subtracting the frequency of dimer/trimer from the frequency of the monomer for the corresponding vibrational mode.
4. I is the computed IR intensity of the dimer/trimer and I_o is the computed IR intensity of the monomer.

Table: ST 7. Summary of the FMO analysis of the dimers and trimers of the methanethiol molecule. (HOMO & LUMO values are in eV)

Conformers of dimer	HOMO	LUMO	Conformers of trimer	HOMO	LUMO
1A	-9.572426	3.754901	2A	-9.657598	3.421017
1B	-9.582222	3.940755	2B	-9.651067	3.881162
1C	-9.617325	3.945925	2C	-9.650795	3.881706
1D	-9.582222	3.939939	2D	-9.650795	3.881706
1E	-9.572698	3.756806	2E	-9.651067	3.979667

Table: ST 8. Summary of second order perturbation theory analysis of fock matrix in NBO analysis
Inter molecular interactions

Dimer					
Conformer	Donor (i)	Acceptor (j)	E(2) (kJ/mol)	E(j) – E(i) (a.u.)	F(i, j) (a.u.)
1A	**Intramolecular interaction in molecular unit 1**				
	σS1-H2	σ*C3-H4	2.10	0.97	0.040
	LP(2) S1	σ* C3-H5	3.66	0.68	0.045
	LP(2) S1	σ* C3-H6	4.07	0.68	0.047
	**Intramolecular interaction in molecular unit 2**				
	σS7-H8	σ*C9-H11	2.14	0.98	0.041
	LP(2) S7	σ* C9-H10	3.22	0.70	0.043
	LP(2) S7	σ* C9-H12	3.95	0.68	0.047
	**Intermolecular interaction of molecular unit 2 to 1**				
	LP(2) S7	σ* S1-H2	4.00	0.48	0.039
1B	**Intramolecular interaction in molecular unit 1**				
	σS1-H2	σ*C3-H4	4.44	1.08	0.062
	LP(2) S1	σ* C3-H5	3.06	1.41	0.059
	LP(2) S1	σ* C3-H6	6.17	1.07	0.073
	**Intramolecular interaction in molecular unit 2**				
	σS7-H8	σ*C9-H11	2.93	1.43	0.058
	LP(2) S7	σ* C9-H10	5.90	1.07	0.071
	LP(2) S7	σ* C9-H12	4.85	1.07	0.064
1C	**Intramolecular interaction in molecular unit 1**				
	σS1-H2	σ*C3-H4	6.12	1.07	0.072
	LP(2) S1	σ* C3-H5	2.95	1.42	0.058
	LP(2) S1	σ* C3-H6	4.64	1.07	0.063
	**Intramolecular interaction in molecular unit 2**				
	σS7-H8	σ*C9-H11	6.12	1.07	0.072
	LP(2) S7	σ* C9-H10	2.95	1.42	0.058
	LP(2) S7	σ* C9-H12	4.64	1.07	0.063
1D	**Intramolecular interaction in molecular unit 1**				
	σS1-H2	σ*C3-H4	4.19	0.69	0.048
	LP(2) S1	σ* C3-H5	2.07	0.99	0.040
	LP(2) S1	σ* C3-H6	3.34	0.68	0.043
	**Intramolecular interaction inmolecular unit 2**				
	σS7-H8	σ*C9-H11	2.15	0.97	0.041
	LP(2) S7	σ* C9-H10	3.09	0.70	0.042
	LP(2) S7	σ* C9-H12	4.27	0.69	0.043
1E	**Intramolecular interaction in molecular unit 1**				
	σS5-H6	σ*C1-H3	3.07	1.41	0.059
	LP(2) S5	σ* C1-H2	5.17	1.06	0.066
	LP(2) S5	σ* C1-H4	5.92	1.06	0.071
	**Intramolecular interaction in molecular unit 2**				
	σS11-H12	σ*C7-H10	3.03	1.41	0.058
	LP(2) S11	σ* C7-H8	4.94	1.08	0.066
	LP(2) S11	σ* C7-H9	5.58	1.07	0.069

	**Intermolecular interaction of molecular unit 2 to 1**				
	LP(2) S11	σ* S5-H6	2.59	0.83	0.042
**Trimer**					
2A	**Intramolecular interaction in molecular unit 1**				
	σS1-H3	σ*C2-H4	2.08	0.97	0.040
	LP(2) S1	σ*C2-H5	4.48	0.69	0.050
	LP(2) S1	σ*C2-H6	2.77	0.69	0.039
	**Intramolecular interaction in molecular unit 2**				
	σS7-H9	σ*C8-H10	2.08	0.97	0.40
	LP(2) S7	σ*C8-H11	4.48	0.69	0.050
	LP(2) S7	σ*C8-H12	2.77	0.69	0.039
	**Intramolecular interaction in molecular unit 3**				
	σS13-H15	σ*C14-H16	2.08	0.97	0.40
	LP(2) S13	σ*C14-H17	4.48	0.69	0.050
	LP(2) S13	σ*C14-H18	2.77	0.69	0.039
	**Intermolecular interaction of molecular unit 1 to 2**				
	LP(2) S1	σ*S7-H9	4.85	0.48	0.043
	**Intermolecular interaction of molecular unit 2 to 3**				
	LP(2) S7	σ*S13-H15	4.83	0.48	0.043
	**Intermolecular interaction of molecular unit 3 to 1**				
	LP(2) S13	σ*S1-H3	4.85	0.48	0.043
2B	**Intramolecular interaction in molecular unit 1**				
	σS1-H9	σ*C2-H4	2.04	0.98	0.040
	LP(2) S1	σ*C2-H3	4.40	0.70	0.050
	LP(2) S1	σ*C2-H5	2.74	0.70	0.039
	**Intramolecular interaction in molecular unit 2**				
	σ S7-H12	σ*C8-H10	2.06	0.98	0.040
	LP(2) S7	σ*C8-H9	2.83	0.69	0.040
	LP(2) S7	C8-H11	4.39	0.69	0.050
	**Intramolecular interaction in molecular unit 3**				
	σ S13-H118	σ*C14-H17	2.05	0.97	0.040
	LP(2) S13	σ*C14-H15	4.49	0.70	0.050
	LP(2) S13	σ*C14-H16	2.68	0.69	0.039
	**Intermolecular interaction of molecular unit 1 to 3**				
	LP(2) S1	σ*S13-H18	5.54	0.48	0.046
	**Intermolecular interaction of molecular unit 2 to 1**				
	LP(2) S7	σ*S1-H6	5.51	0.48	0.046
	**Intermolecular interaction of molecular unit 3 to 2**				
	LP(2) S13	σ*S7-H12	4.81	0.48	0.043
2C	**Intramolecular interaction in molecular unit 1**				
	σS1-H3	σ*C2-H4	2.04	0.98	0.040
	LP(2) S1	σ*C2-H5	2.74	0.70	0.039
	LP(2) S1	σ*C2-H6	4.39	0.70	0.050
	**Intramolecular interaction in molecular unit 2**				
	σS7-H3	σ*C8-H10	2.05	0.98	0.040
	LP(2) S7	σ*C8-H11	4.49	0.70	0.050
	LP(2) S7	σ*C8-H12	2.67	0.69	0.039
	**Intramolecular interaction in molecular unit 3**				

	σS13-H15	σ*C14-H16	2.06	0.98	0.040
	LP(2) S13	σ*C14-H17	4.39	0.69	0.050
	LP(2) S13	σ*C14-H18	2.83	0.69	0.040
	**Intermolecular interaction of molecular unit 1 to 2**				
	LP(2) S1	σ*S7-H9	5.53	0.48	0.046
	**Intermolecular interaction of molecular unit 2 to 3**				
	LP(2) S7	σ*S13-H15	4.80	0.48	0.043
	**Intermolecular interaction of molecular unit 3 to 1**				
	LP(2) S13	σ*S1-H3	5.51	0.48	0.046
2D	**Intramolecular interaction in molecular unit 1**				
	σS1-H3	σ*C2-H4	2.08	0.97	0.040
	LP(2) S1	σ* C2-H5	4.31	0.68	0.049
	LP(2) S1	σ* C2-H6	3.22	0.69	0.042
	**Intramolecular interaction in molecular unit 2**				
	σS7-H9	σ*C8-H10	2.06	0.98	0.040
	LP(2) S7	σ* C8-H11	4.53	0.69	0.050
	LP(2) S7	σ* C8-H12	2.67	0.70	0.039
	**Intramolecular interaction in molecular unit 3**				
	LP(2) S13	σ* C14-H17	4.50	0.71	0.051
	LP(2) S13	σ* C14-H18	2.12	0.71	0.035
	**Intermolecular interaction of molecular unit 1 to 3**				
	LP(2) S1	σ* C14-H16	2.03	0.69	0.042
	**Intermolecular interaction of molecular unit 2 to 1**				
	LP(2) S7	σ* S1-H3	5.00	0.49	0.044
	**Intermolecular interaction of molecular unit 3 to 2**				
	LP(2) S13	σ* S7-H9	5.33	0.50	0.046
2E	**Intramolecular interaction in molecular unit 1**				
	σS1-H3	σ*C2-H4	2.05	0.97	0.040
	LP(2) S1	σ* C2-H5	4.49	0.70	0.050
	LP(2) S1	σ* C2-H6	2.67	0.69	0.039
	**Intramolecular interaction in molecular unit 2**				
	σS7-H3	σ*C8-H10	2.06	0.98	0.040
	LP(2) S7	σ* C8-H11	4.39	0.69	0.050
	LP(2) S7	σ* C8-H12	2.83	0.69	0.040
	**Intramolecular interaction in molecular unit 3**				
	σS13-H3	σ*C14-H16	2.04	0.98	0.040
	LP(2) S13	σ* C14-H17	2.74	0.70	0.039
	LP(2) S13	σ* C14-H18	4.40	0.70	0.050
	**Intermolecular interaction of molecular unit 1 to 2**				
	LP(2) S1	σ* S7-H9	4.80	0.48	0.043
	**Intermolecular interaction of molecular unit 3 to 1**				
	LP(2) S13	σ* S1-H3	5.53	0.48	0.046

Table: ST 9. Absolute values of the S-H vibrational frequencies at different levels of theory. All frequencies are given in cm^{-1}

**S-H vibration mode**	**B3LYP/cc-pVDZ**		**B3LYP/cc-pVTZ**		**B3LYP/cc-pVQZ**		**CCSD/cc-pVDZ**		**Exp.**
	**υ**	**I**	**υ**	**I**	**υ**	**I**	**υ**	**I**	
**1A and 1E** (7,8)	**2599.43**	45.13	2523.39	51.99	2507.78	57.01	2729.78	7.51	**2601**
(1, 2)	2652.25	8.00	2575.97	4.84	2560.67	3.34	2730.36	6.80	
**1B and 1D** (7,8)	2650.52	14.30	2575.09	9.39	2559.79	7.3412	2732.04	16.50	
(1,2)	2655.37	419	2578.79	2.1675	2562.92	0.9326	2734.71	4.83	
**1C** (7,8)	2651.23	21.30	2575.78	0.00	2560.58	0.00	2731.56	26.26	
(1, 2)	2651.29	0.00	2575.81	12.47	2560.61	8.57	2731.57	0.00	
**2A** (1, 3)	**2574.28**	0.49	2616.32	0.13	2620.16	0.12	2718.72	0.61	**2567**
(7, 9)	2583.75	123.46	2621.01	74.80	2624.17	72.92	2723.05	16.00	
(13, 15)	2584.39	123.26	2621.30	74.85	2624.40	72.81	2723.08	16.00	
**2B, 2C, and 2E** (1, 6)	2562.47	17.48	2604.16	24.70	2608.25	30.35	2719.71	6.64	
(13, 18)	2572.37	151.17	2609.30	100.23	2613.22	93.21	2727.13	5.31	
(7, 12)	2580.71	116.24	2621.26	57.99	2625.09	54.17	2728.70	4.16	
**2D** (7, 9)	2580.62	47.99	2615.43	34.07	2617.12	41.74	2720.80	11.86	
(1, 3)	2587.27	114.99	2619.08	80.55	2620.79	77.03	2728.00	6.15	
(13, 15)	2652.02	8.88	2670.44	4.91	2670.14	3.21	2731.23	11.46	

Table: ST 10. Topological parameters for bonds of interacting atoms: electron density (ρBCP), Laplacian of electron density (∇^2 BCP), kinetic electron energy density (G BCP), potential electron energy density (VBCP), total electron energy density (HBCP), estimated interaction energy (E_{int}) at bond critical point (BCP)

**Dimer**							
Confor.	Critical point number	ρ_{BCP} (a.u.)	$\nabla^2 \rho_{BCP}$ (a.u.)	G_{BCP} (a.u.)	V_{BCP} (a.u.)	H_{BCP} (a.u.)	E_{int} (kcal/mol)
1A	19(CH- -S)	0.007675	0.019130	0.004387	-0.003992	0.000395	1.253
	24(SH- -S)	0.002562	0.015372	0.003230	-0.000262	0.000613	0.821
1B	4(CH- -S)	0.005405	0.015802	0.003412	-0.002874	0.000528	0.902
	8(SH- -S)	0.003768	0.011871	0.002417	-0.001867	0.005504	0.586
	13(CH- -S)	0.006043	0.016176	0.003590	-0.003135	0.000454	0.984
1C	9(CH- -S	0.005072	0.014728	0.003130	-0.002578	0.000552	0.809
	4(CH- -S)	0.005072	0.014728	0.003130	-0.002578	0.000552	0.809
1D	4(CH- -S)	0.005407	0.015807	0.003413	-0.002875	0.000538	0.902
	8(SH- -S)	0.003763	0.011860	0.002415	-0.001865	0.000550	0.585
	13(SH- -C)	0.006049	0.016186	0.003592	-0.003138	0.000454	0.985
1E	13(CH- -S)	0.007669	0.019118	0.004384	-0.003989	0.000395	1.252
	15(SH- -S)	0.005124	0.015372	0.003230	-0.002617	0.000613	0.821
**Trimer**							
2A	7((CH- -HC)	0.002358	0.009885	0.001816	-0.001160	0.000655	0.364

	8(SH- -S)	0.008447	0.019123	0.004539	-0.004298	0.000242	1.349
	10(CH- -HC)	0.002368	0.009924	0.001824	-0.001167	0.000657	0.366
	16(CH- -HC)	0.002360	0.009895	0.001818	-0.001162	0.000656	0.365
	17(SH- -S)	0.008451	0.019130	0.004541	-0.004300	0.000241	1.349
	20(SH- -S)	0.008447	0.019123	0.004539	-0.004298	0.000242	1.349
2B	19(SH- -S)	0.009036	0.021120	0.005007	-0.004733	0.000273	1.485
	22(CH- -HC)	0.002678	0.011446	0.002095	-0.001329	0.000766	0.417
	23(SH- -S)	0.007256	0.017466	0.004007	-0.003647	0.000360	1.144
	24(SH- -S)	0.008500	0.020637	0.004812	-0.004464	0.000348	1.401
2C	6(SH- -S)	0.009041	0.021121	0.005008	-0.004736	0.000272	1.486
	14(CH- -HC)	0.002666	0.011395	0.002086	-0.001322	0.000763	0.415
	15(SH- -S)	0.007279	0.017512	0.004019	-0.003660	0.000359	1.148
	16(SH- -S)	0.008502	0.020635	0.004812	-0.004465	0.000347	1.401
2D	7(SH- -S)	0.380863	-12.0882	0.006628	-3.035302	-3.028673	952.341
	10(CH- -HC)	120.6446	666646.6	5.189772	-166672.0	-166666.8	52294140.5
	12(SH- -S)	0.379907	-12.0370	0.006653	-3.022550	-3.015897	948.340
	14(SH- -S)	0.381102	-12.1061	0.006623	-3.039763	-3.033140	953.740
	20(CH- -HC)	0.001891	0.007797	0.001478	-0.001007	0.000471	0.316
2E	6(SH- -S)	0.009030	0.021103	0.005003	-0.004730	0.000273	1.484
	13(CH- -HC)	0.002652	0.011340	0.002075	-0.001314	0.000760	0.412
	14(SH- -S)	0.007253	0.017470	0.004007	-0.003646	0.000361	1.144
	15(SH- -S)	0.008494	0.020619	0.004808	-0.004461	0.000347	1.400

[Note: Topological results also confirms the similarity of the conformers 1A and 1E; 1B and 1D; 2B, 2C, and 2E. But the BCP number is different correspond to each conformer that is why we provide topological results in to separate column.]

Table: ST11. Thermodynamic parameters of different conformers of dimer and trimer of the methanethiol molecule

**Dimer**						
	**Methods**	**ΔE kJ/mol.**	**D_0 kJ/mol.**	**ΔG kJ/mol.**	**ΔH kJ/mol.**	**ΔS kJ/mol.**
**1A and 1E**	B3LYP/cc-pVDZ	-4.54	-1.10	29.87	0.96	-0.10
	B3LYP/cc-pVTZ	-1.87	2.60	42.57	1.53	-0.14
	B3LYP/cc-pVQZ	-1.30	3.10	43.48	1.85	-0.14
	B3LYP/CBS	-0.88	3.46	44.14	2.08	-0.14
	CCSD/cc-pVDZ	-3.76	-0.65	28.67	1.74	-0.09
**1B and 1D**	B3LYP/cc-pVDZ	-4.00	-1.20	28.54	1.44	-0.09
	B3LYP/cc-pVTZ	-1.89	2.39	42.27	1.56	-0.14
	B3LYP/cc-pVQZ	-1.28	2.96	43.25	1.92	-0.14
	B3LYP/CBS	-0.83	3.38	43.97	2.18	-0.14
	CCSD/cc-pVDZ	-4.49	-2.45	27.98	0.06	-0.09
**1C**	B3LYP/cc-pVDZ	-3.03	-1.07	30.36	-0.23	-0.10
	B3LYP/cc-pVTZ	-1.11	2.89	41.77	2.24	-0.13
	B3LYP/cc-pVQZ	-0.58	3.41	42.80	2.54	-0.14
	B3LYP/CBS	-0.19	3.79	43.55	2.76	-0.15
	CCSD/cc-pVDZ	-4.44	-2.37	28.04	-1.56	-0.10
**Trimer**						
**2A**	B3LYP/cc-pVDZ	-13.07	-6.22	-2.16	59.46	0.21
	B3LYP/cc-pVTZ	-6.66	-1.06	3.89	60.49	0.19

	B3LYP/cc-pVQZ	-5.48	-0.14	5.02	59.8	0.18
	B3LYP/CBS	-4.62	0.53	5.84	59.30	0.17
	CCSD/cc-pVDZ	-9.33	-2.93	1.69	62.62	0.20
**2B, 2C and 2E**	B3LYP/cc-pVDZ	-15.07	-7.97	-4.04	57.99	0.21
	B3LYP/cc-pVTZ	-8.85	-3.00	1.81	58.33	0.19
	B3LYP/cc-pVQZ	-7.69	-2.04	2.93	58.23	0.19
	B3LYP/CBS	-6.84	-1.34	3.75	58.16	0.19
	CCSD/cc-pVDZ	-10.27	-3.3	0.99	64.8	0.21
**2D**	B3LYP/cc-pVDZ	-12.93	-6.38	-1.95	59.12	0.20
	B3LYP/cc-pVTZ	-7.59	-2.13	3.07	57.96	0.18
	B3LYP/cc-pVQZ	-6.48	-1.24	4.12	56.54	0.18
	B3LYP/CBS	-5.67	-0.59	4.89	55.50	0.18
	CCSD/cc-pVDZ	-11.61	-5.19	-0.45	60.92	0.21

Note: - Z-matrix of the optimized geometries of the methanethiol cluster at CCSD/cc-pVDZ level of theory

Monomer

Symbolic Z-matrix:
Charge = 0 Multiplicity = 1

C	0.04791	1.15862	0.
H	1.09202	1.4609	0.
H	-0.43099	1.55252	0.89181
H	-0.43099	1.55252	-0.89181
S	0.04791	-0.66753	0.
H	-1.28404	-0.83712	0.

Dimer

1A

Symbolic Z-matrix:
Charge = 0 Multiplicity = 1

S	1.80964	0.63013	-0.3411
H	0.65051	0.01184	-0.7034
C	2.57623	-0.81531	0.49435
H	3.54598	-0.47023	0.88062
H	1.96332	-1.16351	1.33802
H	2.74918	-1.63894	-0.21299
S	-2.04283	-0.64698	-0.30887
H	-1.64863	-1.3836	0.76242
C	-2.10242	0.97835	0.54867
H	-1.10232	1.27736	0.89292
H	-2.45476	1.70112	-0.20101
H	-2.8151	0.95726	1.38472

1B

Symbolic Z-matrix:
Charge = 0 Multiplicity = 1

S	-1.94593	0.6054	-0.31355
H	-1.2726	0.13319	-1.39453
C	-2.00087	-0.97853	0.61877
H	-0.9868	-1.36227	0.79656
H	-2.47626	-0.74709	1.58271
H	-2.60956	-1.72662	0.09154
S	1.85717	-0.56872	-0.34718
H	3.13733	-0.97801	-0.14867
C	1.96219	1.00266	0.5985
H	2.67806	1.69767	0.13747
H	0.95192	1.43407	0.55488
H	2.23016	0.8174	1.64818

1C

Symbolic Z-matrix:
Charge = 0 Multiplicity = 1

S	2.10164	-0.49592	-0.28156
H	3.29138	-0.47535	0.36027
C	1.76382	1.30089	-0.1647
H	2.5013	1.88537	-0.73825
H	0.76189	1.45473	-0.59647
H	1.74802	1.63661	0.88475
S	-2.10164	0.49592	0.28156
H	-3.29138	0.47535	-0.36027
C	-1.76382	-1.30089	0.1647
H	-0.76189	-1.45473	0.59647
H	-2.5013	-1.88537	0.73825
H	-1.74802	-1.63661	-0.88475

1D

Symbolic Z-matrix:
Charge = 0 Multiplicity = 1

S	1.85594	-0.5685	-0.34799
H	3.13406	-0.98249	-0.14592
C	1.96298	1.00135	0.59979
H	2.68189	1.69511	0.14153
H	0.95396	1.43556	0.55381
H	2.22763	0.81431	1.65001
S	-1.9441	0.60542	-0.31458
H	-1.26825	0.13087	-1.39295
C	-2.00269	-0.97698	0.62
H	-0.98952	-1.36162	0.80102
H	-2.48008	-0.74354	1.58247
H	-2.61096	-1.72523	0.0925

1E

Symbolic Z-matrix:
Charge = 0 Multiplicity = 1

C	-2.57665	-0.81521	0.49427
H	-1.96322	-1.16416	1.33724
H	-3.54582	-0.4696	0.88148
H	-2.75061	-1.63829	-0.21344
S	-1.8096	0.63023	-0.34102
H	-0.65045	0.01179	-0.70293
C	2.10305	0.9784	0.54851
H	1.10301	1.27788	0.89247
H	2.81559	0.95686	1.38464
H	2.45592	1.7008	-0.20126
S	2.04264	-0.64717	-0.30883
H	1.64847	-1.38344	0.76272

Trimer

2A

Symbolic Z-matrix:
Charge = 0 Multiplicity = 1

S	-0.99556	2.20104	-0.66784
C	-0.90035	2.33255	1.15716
H	0.23967	1.67632	-0.84161
H	-1.84999	2.7809	1.49278
H	-0.07092	2.98893	1.46657
H	-0.79177	1.34266	1.63044
S	-1.40788	-1.9623	-0.66834
C	-1.57205	-1.94617	1.15642
H	-1.57049	-0.63011	-0.84199
H	-1.48674	-2.99295	1.49177
H	-2.5554	-1.55549	1.46452
H	-0.76937	-1.35794	1.63113
S	2.40396	-0.23778	-0.66783
C	2.47145	-0.38803	1.15696
H	1.33197	-1.04516	-0.84184
H	3.33465	0.21	1.4927
H	2.62557	-1.43485	1.46508
H	1.56011	0.01217	1.63127

2B

Symbolic Z-matrix:
Charge = 0 Multiplicity = 1

S	2.15686	-0.24412	0.81484
C	3.29549	-0.22755	-0.62756
H	3.85275	0.7184	-0.67634
H	4.00463	-1.05285	-0.47193
H	2.7518	-0.39595	-1.56763
H	1.35992	0.78618	0.4058
S	-0.7343	2.17527	-0.66729
C	-1.67891	2.57463	0.85727
H	-1.41354	1.89865	1.68237
H	-1.39973	3.60103	1.13449
H	-2.76131	2.53824	0.66999
H	-1.18376	0.89495	-0.81056
S	-1.19989	-1.87841	-0.7524
C	-1.99519	-2.44007	0.80599
H	-1.50933	-3.34821	1.18962
H	-1.97863	-1.65131	1.57111
H	-3.03979	-2.67304	0.55516
H	0.02596	-1.60203	-0.21881

2C

Symbolic Z-matrix:
Charge = 0 Multiplicity = 1

S	2.15661	-0.23877	0.81419
C	3.29516	-0.21823	-0.62824
H	1.35619	0.78882	0.40511
H	4.00734	-1.04088	-0.47247
H	2.75197	-0.38885	-1.5682
H	3.84891	0.72976	-0.67726
S	-1.19639	-1.88139	-0.75277
C	-1.98343	-2.44924	0.80758
H	0.03044	-1.60139	-0.22335
H	-3.02818	-2.6854	0.56038
H	-1.4928	-3.35647	1.18728
H	-1.9666	-1.66211	1.57436
S	-0.74138	2.17324	-0.66733
C	-1.6837	2.57391	0.85832
H	-1.18931	0.89206	-0.80769
H	-1.40581	3.60135	1.13296
H	-2.7664	2.53521	0.67326
H	-1.41545	1.90002	1.68421

2D

Symbolic Z-matrix:
Charge = 0 Multiplicity = 1

S	-0.39682	2.48325	-0.53966
C	-0.44059	2.04845	1.2404
H	-1.02636	1.36579	-0.96994
H	0.12576	2.83	1.77327
H	-1.47443	2.03837	1.62328
H	0.04011	1.07407	1.42486
S	-1.61014	-1.48512	-0.76941
C	-2.54409	-1.48019	0.80749
H	-0.37724	-1.49397	-0.21205
H	-3.61334	-1.44953	0.54105
H	-2.34864	-2.39613	1.38804
H	-2.30325	-0.59241	1.41418
S	2.24687	-0.99628	0.69953
C	2.46417	-0.33931	-0.99841
H	2.78251	-2.21643	0.47034
H	1.97771	0.6495	-1.00949
H	3.53171	-0.22626	-1.2462
H	1.9699	-0.98631	-1.74148

2E

Symbolic Z-matrix:
Charge = 0 Multiplicity = 1

S	-1.64106	-1.3988	-0.61091
C	-2.91442	-1.09152	0.67029
H	-0.57594	-1.39849	0.22409
H	-3.88318	-1.04386	0.14646
H	-2.94725	-1.91414	1.40263
H	-2.74314	-0.13374	1.18757
S	0.1696	2.37109	-0.71491
C	-0.60136	2.26871	0.94406
H	-0.47649	1.29884	-1.22732
H	-0.15151	3.06854	1.55526
H	-1.6895	2.43499	0.88804
H	-0.39167	1.29556	1.41746
S	2.14075	-0.82285	0.87207
C	2.3516	-1.45374	-0.83572
H	1.94812	0.46814	0.51538
H	2.44333	-2.54987	-0.76236
H	1.47749	-1.20916	-1.46125
H	3.26615	-1.0486	-1.29765
